# Topological computation based on direct magnetic logic communication

**DOI:** 10.1038/srep15773

**Published:** 2015-10-28

**Authors:** Shilei Zhang, Alexander A. Baker, Stavros Komineas, Thorsten Hesjedal

**Affiliations:** 1 Department of Physics, Clarendon Laboratory, University of Oxford, Oxford, OX1 3PU, United Kingdom; 2 Department of Mathematics and Applied Mathematics, University of Crete, 71409 Heraklion, Crete, Greece

## Abstract

Non-uniform magnetic domains with non-trivial topology, such as vortices and skyrmions, are proposed as superior state variables for nonvolatile information storage. So far, the possibility of logic operations using topological objects has not been considered. Here, we demonstrate numerically that the topology of the system plays a significant role for its dynamics, using the example of vortex-antivortex pairs in a planar ferromagnetic film. Utilising the dynamical properties and geometrical confinement, direct logic communication between the topological memory carriers is realised. This way, no additional magnetic-to-electrical conversion is required. More importantly, the information carriers can spontaneously travel up to ~300 nm, for which no spin-polarised current is required. The derived logic scheme enables topological spintronics, which can be integrated into large-scale memory and logic networks capable of complex computations.

The concept of topology in physics not only describes matter and its excitations from an insightful perspective, but also brings about inspirations for practical applications^1^,^2^,^3^. In the field of spintronics, where commonly uniformly aligned spins are exploited as a state variable for storage bits^4^, superparamagnetism limits the scaling-down of the magnetic domains^5^,^6^. However, there are increasing efforts trying to make use of nontrivial spin textures for nonvolatile memory^2^,^7^,^8^. The underlying argument that separates ‘trivial’ from ‘non-trivial’ systems arises from the topological description of the field configuration^9^. Magnetic skyrmions, for instance, are a type of magnetisation configuration which can be thought of as a ‘hedgehog’-like spin distribution being projected onto a two-dimensional plane^2^,^10^.

Starting with magnetic bubbles observed in ferromagnetic films with perpendicular easy-axis anisotropy, topological magnetic solitons^11^,^12^ have been an attractive topic due to the rich physics associated with their static and dynamical properties. Magnetic skyrmions are essentially homotopy-equivalent to magnetic bubbles^1^,^2^. Magnetic vortices were extensively studied in systems with in-plane anisotropy^2^.

In a continuum approximation for a ferromagnetic film the reduced magnetisation 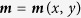 is a function of space but has a constant magnitude ***m***^2^ = 1. A spin configuration is characterized by a topological charge called the skyrmion number and defined by


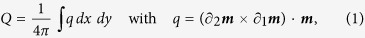


where *q* is a topological density which also plays the role of a local vorticity^13^. The topological charge *Q* takes integer values when the magnetisation at spatial infinity is uniform. A magnetic soliton with skyrmion number *Q*  ≠ 0 is called ‘topologically nontrivial’. Its coherence and robustness is partially protected because the magnetisation configuration cannot be annihilated or destroyed by a gradual (continuous) change to a uniform or trivial *Q* *=* 0 configuration. Therefore, a topological soliton has a particle-like character and can be considered a more robust information carrier than a trivial domain.

Introducing the concept of topology to magnetic memory not only means that all advantages that magnetic memory has over conventional charge-based memory are inherited, but also that much lower current densities are required to induce the collective motion of information^14^. It therefore offers excellent compatibility with the proposed, three-dimensional (3D) racetrack memory concept^15^,^16^. In the topological memory scheme, binary information ‘0’/‘1’ that is encoded by the magnetisation orientation (e.g., up and down) is replaced by the topological number, *Q*, which can take integer or fractional values. One of the most promising concepts is skyrmion-based racetrack memory. Figure 1a illustrates the 3D memory structure in which the topological charge substitutes the magnetisation state as the binary state variable. The read-out of information may be implemented by measuring the local Hall effect^17^, while writing is performed by locally nucleating/annihilating topological charge.

While there is a clear vision for topological memory, the implementation of topological logic remains lacking despite it being an essential component for the development of ‘topological spintronics’. With the introduction of topological memory bits, the existing magnetic logic proposals, e.g., domain wall^16^ or magnetic tunnel junction logic^18^, are no longer possible^19^. The usual approach to achieve logic functionality would be to use topology only as an internal variable, and to translate topology into a charge signal by measuring magnetotransport quantities such as the Hall voltage or magnetoresistance^20^,^21^. The subsequent circuitry would then transfer the charge-based information to the conventional transistor-based logic gate. Following the computation, an additional device is also required to write the result back into the topological memory bit. This would make logic operations inefficient in time, space, and energy.

The challenge can be addressed by redesigning the principles of logic operations on the lowest level, i.e., by translocating the stored spin texture directly to the assigned logic gate for computation. In such a scheme, the continuous topology-to-charge conversions can be omitted, which constitutes a significant step towards an all-topological circuit, in analogy to the all-spin-circuit concept^4^. Here, we numerically demonstrate that direct logic communication can be realised for a type of magnetic semi-topological object, the propagating vortex-antivortex (VA) pair. By utilising its unique dynamical features, an effective information transfer channel can be established for logic operations.

## Results

### Modelling and analytical solution

We consider a ferromagnetic permalloy (Py) thin film system. In the continuum approximation the local magnetic moment 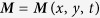 has a fixed length of 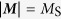, where *M*_S_ is the (constant) saturation magnetisation. The dynamics follows the Landau-Lifshitz-Gilbert (LLG) equation in a phenomenological description. This reads, in rationalized form^22^,


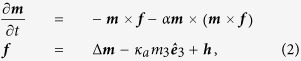


where 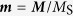 is the reduced magnetisation with unit length 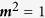, and *α* is the damping constant. The effective field ***f*** contains three terms: the exchange field given by the Laplacian of the magnetisation, an easy-plane anisotropy field with dimensionless constant 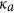 and 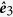 the unit vector in the out-of-plane direction, and the dimensionless magnetostatic field ***h*** = ***H***/*M*_*s*_ where ***H*** is the magnetostatic field in SI units. Distance is measured in units of the exchange length 
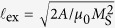
 where *A* is the exchange constant, and time is measured in units of 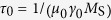 where *γ*_0_ is the gyromagnetic ratio. For detailed values of the simulation parameters, see Methods. The effective field ***f*** can be derived from the functional derivative of the energy *E*:


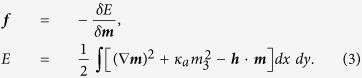


Axially symmetric vortices are conveniently described using the spherical parametrisation for the vector ***m***:


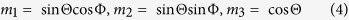


and they are of the form 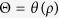 and 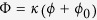, where 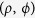 are polar coordinates, 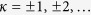 will be denoting the winding number, and *ϕ*_0_ is an arbitrary constant expressing the azimuthal ‘phase shift’. We find *θ*(*ρ*) solving the LL equation and imposing the boundary conditions 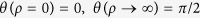; then an axially symmetric vortex solution reads


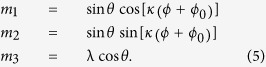


**Figure 1:**
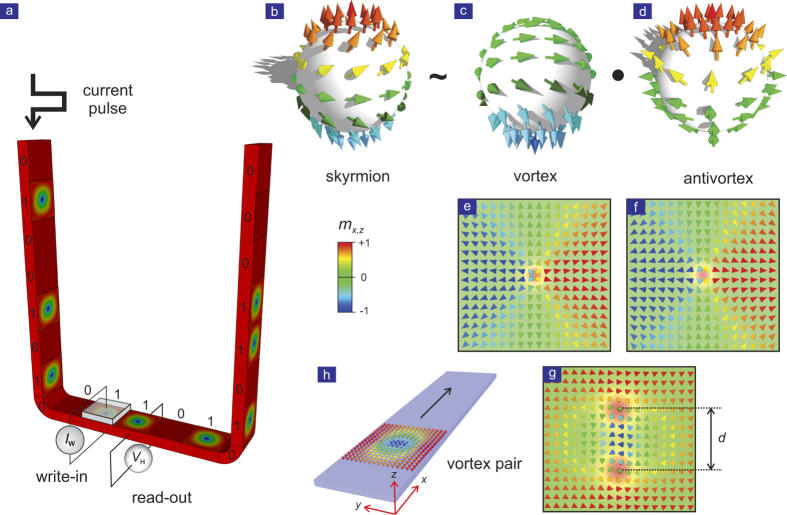
Conceptual scheme of the topological objects and 3D topological racetrack memory. (**a**) In a skyrmion-based racetrack memory, the binary variables, i.e., magnetic domains with opposite magnetisation orientation, are replaced by topological charge. Thus the binary ‘1’ is encoded by a local domain with a topological number *Q* ≠ 0, while ‘0’ is encoded by a uniform domain with *Q* = 0. A current is applied to exert spin transfer torque in order to shift the register, i.e., move the whole domain pattern. Writing can be implemented by locally nucleating/deleting the topology domain by electrical means. Reading can be realised by measuring the local topological Hall effect. (**b**) Stereographic-projection view of the magnetisation distribution of a single magnetic skyrmion, which is homotopically equivalent to a magnetic vortex with its core magnetisation pointing down (**c**), conjugated with an antivortex with its core pointing up (**d**). The corresponding real-space spin textures of the single vortex, single antivortex, and a conjugated pair are shown in (**e**), (**f**), and (**g**) respectively. (**h**) Simulation coordinates for the vortex-antivortex pair dynamics.

The value 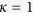 corresponds to a single vortex while for *κ* = −1 we have an antivortex. The polarity λ = ±1 governs the out-of-plane component of the magnetisation: λ = 1 corresponds to the *m*_3_ component in the vortex core pointing up, and λ = −1 to the vortex core pointing down. Figure 1e,f, illustrates the vortex spin mappings obtained from equation (5) for *ϕ*_0_ = 0. The skyrmion number (1) of a single vortex is a half-integer


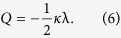


The magnetisation can also be expressed by its stereographic projection from the north pole onto the equatorial plane, which is given by the complex variable


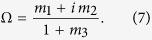


For the axially symmetric vortex in Eq. (5) we have the form


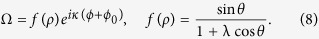


This representation is convenient for the construction of multi-vortex configurations. An ansatz for a vortex-antivortex pair where the vortex is placed at position 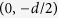 and the antivortex at 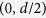 is the following^12^:


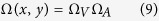


where


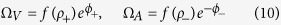


and


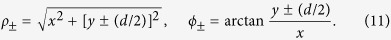


From the magnetic memory perspective, more degrees of freedom are obtained by breaking a topological object into two halves. The direct consequence is that one can have four different combinations of conjugating two *Q* = 1/2 objects, namely, a vortex-antivortex pair. There are two kinds of a vortex-antivortex pair. If the vortex has negative and the antivortex positive polarity then the skyrmion number of the pair is 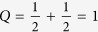 as can be inferred from Eq. (6). Therefore, the topology of a skyrmion can be homotopically decomposed into a vortex and an antivortex as schematically illustrated in Fig. 1b–d. If the vortex and the antivortex have the same polarity then the skyrmion number of the pair is 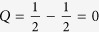 and they will be propagating along parallel lines^12^. We will focus on the latter vortex-antivortex pairs in the following.

### Dynamics and simulation results

The main results on the dynamics of vortices can be better understood if we momentarily focus on the conservative LL equation, i.e., Eq. (2) with *α* = 0. A relation for the time evolution of the local vorticity *q* is obtained by application of the LL equation and proves to be fundamental for the dynamics^13^,^22^:


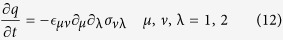


where 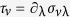 is the ‘force density’ introduced by Thiele^23^,^24^. The components of the tensor *σ* for the interactions contained in the effective field of Eq. (2) have been obtained in ref. 22. The dynamics of Eq. (12) can be interpreted as the temporal rate-of-change of the local vorticity being equal to the divergence of the force density of the system. An immediate consequence of Eq. (12) is that the total vorticity, 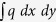, and thereby the skyrmion number *Q* are conserved. Furthermore, the linear momentum 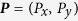 is defined by:


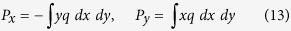


and it is also conserved by virtue of the second order spatial derivatives on the right-hand-side of Eq. (12). The above are rigorous conservation laws in an infinite film.

The detailed dynamics of VA pairs within the LL equation is studied in refs 12, 25 for the easy-plane model with 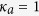 and ***h*** = 0. The VA pair with opposite polarities (*Q* = 1) presents rotational motion, while the VA pair with same polarities (*Q* = 0) undergoes Kelvin motion, i.e., the vortices propagate along parallel lines. The latter behaviour is familiar from ordinary vortex-antivortex pairs in a fluid and it is similar to the motion of an electron-positron pair in a magnetic field^13^,^26^. Since the Kelvin pair is composed of topological objects (vortex and antivortex) but has an overall skyrmion number *Q* = 0, it has been called a ‘semitopological soliton’. From an application point of view, Kelvin motion is of great interest as spontaneous displacement suggests an efficient way for transferring information.

Let us assume a *Q* = 0 Kelvin pair with the vortex and antivortex separated by a distance *d* in the *y*-direction. The local vorticity *q* then has two peaks around the points 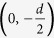 and 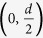, where the cores of the vortex and the antivortex are centred. Consequently, the linear momentum defined in Eq. (13) has components


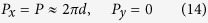


and the VA pair propagates in the *x* direction.

The velocity *v* of the pair when the vortex and antivortex are well separated can be estimated via a virial relation:


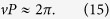


Combining Eq. (14) and (15) one can estimate the velocity


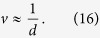


The group-velocity relation *v* = *dE*/*dP* is combined with Eq. (15) to give for the energy of a vortex pair


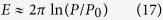


where *P*_0_ is an undetermined integration constant.

A numerical calculation of VA pairs propagating in a steady-state confirms the above results for velocities 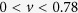. For velocities in the range 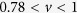 the vortex and antivortex overlap and the topological features are not apparent. As 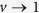 the VA pair annihilates into the ferromagnetic background.

We have performed micromagnetic simulations of vortex dynamics in a thin film strip measuring 1000 × 250 × 2 nm^3^ (cf. Fig. 1h). The initial state is a *Q* = 0 Kelvin pair constructed using ansatz (9) with the vortex and antivortex separated by a distance *d* in the *y*-direction, and situated at one end of a strip. We take full account of geometrical confinement (boundary conditions), magnetostatic energy (dipole-dipole interaction), and damping effects (LLG dynamics).

For the interpretation of simulation results we will use as a guide the results of ref. 12. Since, in the limit of an infinitely thin film the magnetostatic field energy is approximated by an easy-plane anisotropy energy, the model with magnetostatic field and 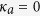 used in the simulations in the present work is quantitatively similar to the model with ***h*** = 0 and 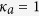 studied in ref. 12. Furthermore, the theory is based on general arguments of the vortex topology rather than on the detailed interactions included in the Landau-Lifshitz model. Therefore, the key qualitative and quantitative features of the VA pair behaviour should remain the same.

**Figure 2:**
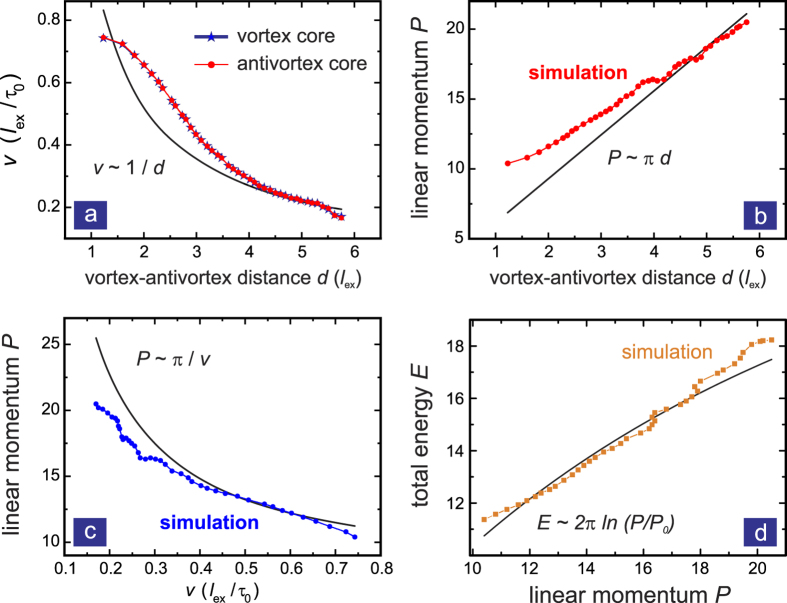
Simulation results for a travelling vortex-antivortex pair. (**a**) The velocity of vortex and antivortex as a function of the distance *d* between them. Initially the vortex and antivortex cores have large *d* and travel with low velocity. They attract each other during propagation, whereby their propagation velocity increases. The vortex and antivortex overlap (at *d* ~ 1) when *v* ~ 0.78. The solid line shows the analytical relationship, *v* − *d*, of Eq. (16). (b) and (c) shows the *P* − *d* and *P* − *v* relationships respectively, which describe the same behaviour as in (**a**). (**d**) *E* − *P* dispersion relation for the same process. We set *P*_0_ = 1.35 obtained from fitting.

The distance *d* between vortex and antivortex and the velocity *v* of the propagating pair are obtained by tracking the vortex cores separately at every time. Correspondingly, the local vorticity and linear momentum are calculated from the magnetisation pattern as it evolves during the simulation. Figure 2 shows the key results of the VA pair dynamics. As the pair starts propagating along the *x*-direction, vortex and antivortex attract each other and *d* is reduced. According to the *v*-*d* relation in Eq. (16) the travelling pair gets accelerated. The more it accelerates, the closer the vortex and antivortex cores get, and vice versa. This iteration terminates when *v* approaches the value 0.78. At this point the pair annihilates rapidly. Figure 2a–d show the relationships of *v*-*d*, *P*-*d*, *P*-*v* (Eqs. (14), (15) & (16)) and *E*-*P* (Eq. (17)), respectively. The numerical results generally follow the trends predicted by the ideal analytical solution, however, deviations are observed. These are mainly due to the geometrical confinement.

We would like to point out that effects of surface roughness, film thickness variations, stripe width variation, and boundary roughness as a result of the lithography process have not been taken into account. As has already been observed, e.g., from the motion of skyrmions^27^ and domain walls in magnetic racetracks^28^, it is to be expected that these effects will change the details of the VA motion as well. A direct consequence of geometrical variations of the channel width are fluctuations of the VA pair velocity along the path. It will be interesting and important to study how pinning defects affect the VA motion.

### Boundary effect and antivortex absorption

**Figure 3:**
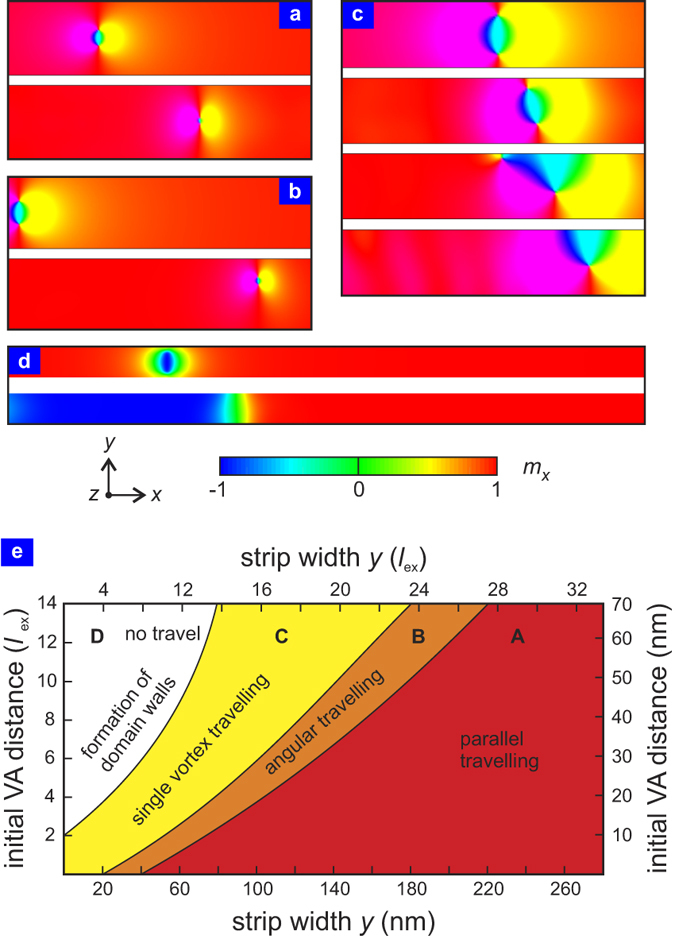
Phase diagram and illustration of the four different VA pair dynamics modes. (**a**) The VA pair propagates unaffected through the structure if the boundary does not influence the motion (compare top and bottom panels). If boundary effects are significant (**b**), the VA pair is attracted to one of the boundaries. (**c**) In case of stronger boundary effects, the antivortex is attracted by the boundary, dragging the vortex up as well. After the antivortex is annihilated, the vortex remains moving through the structure. (**d**) For very strong boundary effects, both vortex and antivortex are ‘absorbed’ by the boundary immediately after they begin to propagate, leading to the formation of domain walls. All images show the projection of the magnetisation vector along the *x*-direction. The contrasts in (**a–c**) are scaled to enhance the details; (**d**) is to scale and the colour code is shown below. (**e**) Phase diagram showing the parameter space in terms of strip width (*y*-axis) and initial VA distance (*y*-axis) for the four different travelling modes. The length of the strip is kept as 1000 nm, and the film thickness at 2 nm. Both exchange-length and real-space units are shown.

The most important way to alter the motion of a VA pair is through boundary effects. In general, antivortices tend to be attracted to the edge of the ferromagnetic object. If they come sufficiently close to the edge, as shown in Fig. 3c, they are absorbed. Since the vortex and antivortex in a VA pair move together, this effect can be used to ‘pull’ the vortex as well. Examples of the effect of the boundary conditions are illustrated in Fig. 3, showing that it is possible to curve the trajectory of the VA pair.

In general, the system exhibits four different modes of vortex motion, depending on the initial VA and the strip width *y*. The corresponding phase diagram illustrating the stability range of each phase of motion is shown in Fig. 3e. If the strip is wide and the initial VA distance large, the VA pair stays intact and both vortex and antivortex travel unperturbed and in parallel along the strip (parallel travelling). For narrower strips, the boundary effect sets in and the pair is attracted to one of the boundaries as it travels along the strip (angular travelling). For even narrower strips, the antivortex is absorbed by one of the boundaries, leaving only the vortex behind. An intriguing result of the boundary effect is that the remaining vortex continues travelling along the strip. The antivortex can therefore be considered as a ‘transporter’ for the binary information stored in the vortex. In the case that the VA pair is placed in a strip of width comparable to *d* no travel is possible. Rather, the VA pair annihilates immediately, releasing a pair of counter-propagating domain walls, as shown in Fig. 3.

**Figure 4:**
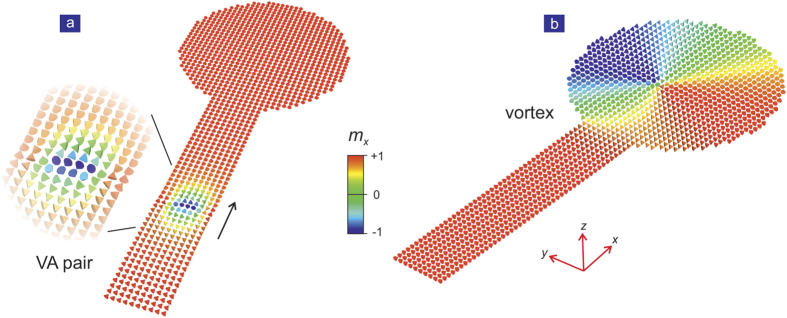
Snapshots of the magnetisation distribution during the a VA travelling process in a terminal device. (**a**) The simulation object is a rectangular strip measuring 500 nm × 125 nm, connected to a disk with a diameter of 300 nm. The initial VA pair distance is 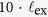, i.e., ~50 nm. The VA pair is created near the end of the strip. The VA will spontaneously travel along the *x*-axis during the relaxation process. (**b**) Due to the boundary confinement, the antivortex is ‘absorbed’ by the upper boundary before entering into the terminal (disk-shaped region), leaving only single vortex, which continues to travel. Finally the vortex is trapped in the terminal, where it remains as a nonvolatile bit.

Moreover, as shown in Fig. 4, the motion of an isolated vortex can be halted and stabilized by the introduction of a disk at the end of the strip, which is termed ‘terminal’. This effectively traps the vortex. After several oscillation within the disk, the vortex remains static. A diagram of such a structure is shown in Fig. 4; the initially travelling VA pair is shown in Fig. 4a and the trapped vortex in Fig. 4b.

## Discussions

### Direct information transfer

The implications of the VA terminal structure for trapping a single vortex are rather significant for spintronic applications. As previously discussed, a single *Q* = 1/2 vortex and a trivial *Q* = 0 ferromagnetic domain can be used as the binary states. In the travelling mode (cf. type **C** in Fig. 3c,e), a vortex (logic ‘1’) can spontaneously travel with the assistance of its ‘antiparticle’, the antivortex, in a controlled way. Consequently, the information is transferred and written into the terminal disk, which can be regarded as a memory cell. On the other hand, the uniform ferromagnetic state (logic ‘0’) will also ‘transfer’ its information to the terminal as the ferromagnetic background throughout the media has *Q* = 0. The whole procedure, i.e., read-out of the information from one cell, transfer of information, and then writing into another cell can be completed within the same material. No extrinsic electrical interaction is needed to read or transfer the information, thereby achieving direct logic communication.

**Figure 5:**
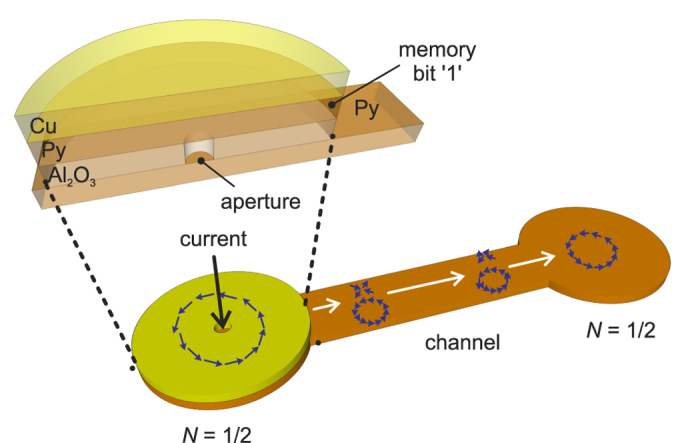
2-terminal fan-out structure for direct information transfer. The bottom channel is based on the structure shown in Fig. 4 and conducts the vortex to the terminal on the end. To generate a VA pair, a heterostructure has to be fabricated at the other end of the structure. The stack consists of Al_2_O_3_ (5 nm)/Py (2 nm)/Cu (5 nm) (from bottom to top), patterned into a disk with a diameter of 300 nm. This structure forms the other terminal which stores the initial binary data in its centre Py layer (*N* = 1/2 vortex for ‘1’, *N* = 0 for ‘0’). The top Cu layer serves as a conducting electrode. When current is applied from the top, the 20–50 nm diameter aperture in the centre of the Al_2_O_3_ layer will effectively aid the generation of a VA pair, in case a vortex is stored in the middle Py layer. No VA pair is formed if the magnetisation in the Py layer is uniform. In this way information stored in the left terminal can be fanned-out to the right terminal.

Furthermore, the design of a topological fan-out structure is proposed. As shown in Fig. 5 symmetric 2-terminal storage cell can be obtained by connecting two VA terminal structures, resembling a dog-bone. The width of the channel satisfies the conditions for the travelling mode *C*, so that only single vortices can pass the channel. At one end of the structure, an injector consisting of a multilayered Al_2_O_3_/NiFe/Cu structure is fabricated on top of the terminal disk. A 20–50 nm diameter aperture is fabricated in the middle of the insulating Al_2_O_3_ layer. The initial information is stored in the Py layer in the middle (*Q* = 1/2 vortex logic ‘1’, *Q* = 0 ferromagnetic state logic ‘0’). It has been demonstrated in simulations that a VA pair may be generated in the bottom Py layer by applying a current to the top Cu electrode^29^. The current picks up the polarity (spin-polarisation) when passing through the middle Py layer, then nucleate a topological pattern in the bottom terminal disk after exiting the aperture. The type of the topology (either a VA pair or no pattern) that is generated in the bottom Py disk depends on the topological state of the middle Py layer. If a VA pair is nucleated, it will spontaneously propagate towards the end of the channel. As the width of the channel is constructed to support the travelling mode *C*, the antivortex will be eventually absorbed by the boundary. Therefore, the binary information ‘1’ (vortex) is written into the other terminal. On the other hand, if no VA pair is generated, the other terminal will stay at binary state ‘0’.

The fan-out device essentially copies the information stored in one cell, and fans it out to another cell that is more than ~300 nm away. The whole process takes less than 3 ns, and occurs inherently within the same media. This effectively realises the transfer of topological information from one bit to the next without the need to know the original state of the bit.

### Direct logic computation

**Figure 6:**
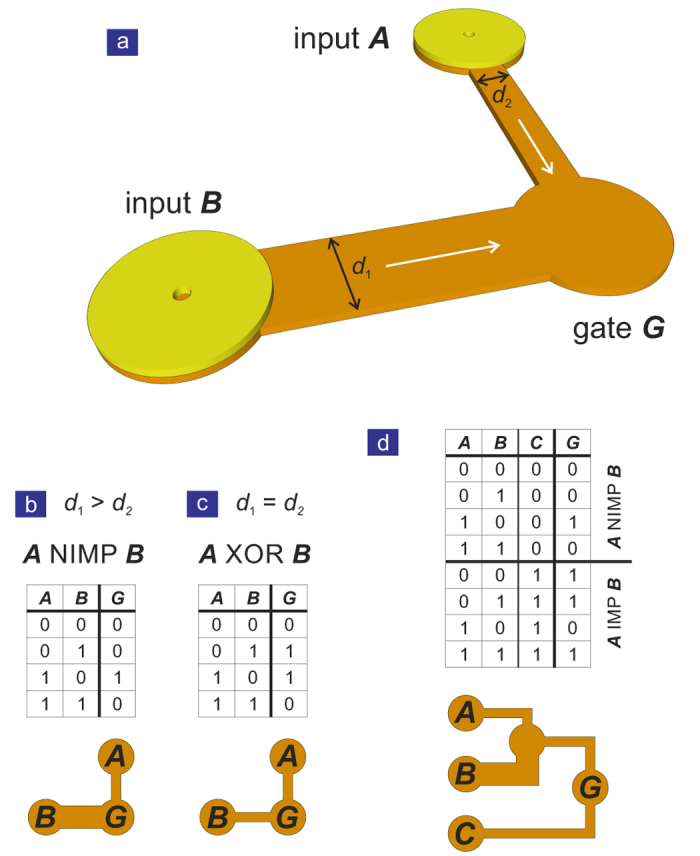
Self-computing logic gate. (**a**) Based on the 2-terminal fan-out structure, different logic functionalities can be achieved by joining two structures in a common terminal (gate *G*). The length and width of the two input channels are tuned to either allow a VA pair or a single vortex to complete the journey. The result of the ensuing three-vortex interaction depends on the arrival time and polarity of the VA pairs, allowing the formation of logic gates such as the NIMP gate (**b**) or the XOR gate (**c**). (**d**) By combining two 2-input gates into a 3-terminal structure, the control bit *C* can be used to achieve the NIMP or IMP gate as shown in the truth table (for details see Supplementary Material).

A direct logic device is realised by joining two 2-terminal structures, each able to carry a VA pair, and sharing a common end terminal (see Fig. 6a). The interaction of vortices with VA pairs can be modelled analytically and numerically, and has well-defined outcomes out of which logic operations can be constructed. Binary information is stored in the terminals and can be converted into VA pairs by application of a spin-polarised current as illustrated in Fig. 5 and discussed above.

The first element in the VA logic toolbox is the tuning of the channel width, whereby either a vortex or a VA pair arrives at the terminal. What constitutes whether a channel is narrow or wide for a given VA pair distance is the channel length, and thus the form factor of the channel. For example, in relation to a travel distance of ~300 nm, a narrow channel is <140 nm and a wide channel >200 nm.

An IMP gate, or ‘material implication’ 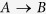 means *A* implies *B*, which is equivalent to 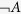 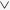 *B*. The concept was introduced by Whitehead and Russell^30^ early last century and the IMP operation, in conjunction with the FALSE operation (the W-W logic gate), forms a computationally complete logic basis^31^, required for arbitrary logic computations. This alternative, yet equivalent set of fundamental logic operations has seen a recent revival in the context of memristor-based logic^32^,^33^.

The second element in the toolbox is the basic logic gate. Figure 6a shows the 2-input logic gate which consist of two 2-terminal fan-out structures sharing the same terminal (termed gate *G*). By adjusting the widths of the channels, several functions can be achieved as illustrated in Fig. 6b–d. In principle, there are three distinct combinations of narrow (N) and wide (W) input channels making up a 2-input element, which exhibit three dynamic magnetisation scenarios. In case of the N-N element, when two vortices arrive at the gate *G*, both of them will decay (→0). In case only one vortex arrives, it will be trapped in the arena (→1). This behaviour is that of a XOR gate and the truth table is shown in Fig. 6c. For the combination of N and W channels, the presence of a VA pair in the W channel will always lead to a decay of the topological objects in the terminal (→0). The only scenario in which a vortex is trapped in the gate is when W is not carrying a VA pair and N is supplying a vortex. The truth table of this NIMP gate logic element is shown in Fig. 6b. The combination of two W channels resets the system and always returns a 0 (not shown). The cascading of the basic elements, e.g., (N-W)-N shown in Fig. 6d, allows for more complex functions. The input *C* acts as a control bit and allows for an inversion (NOT) of the NIMP gate, yielding an IMP gate. Here, material implication (and the FALSE gate) are simply realized by a set of 2-input logic gates. As for the memristor, three gates are sufficient to execute a NAND operation, and as the logic values resulting from the operation are non-volatile, the presented IMP logic is ‘stateful’ combining logic and memory functionalities. The NAND operation, on the other hand, is a universal operation in the sense that arbitrary Boolean logic functions can be achieved through a combination of NAND gates alone. More information about the logic gates used in this work can be found in the Supplementary Material.

**Figure 7:**
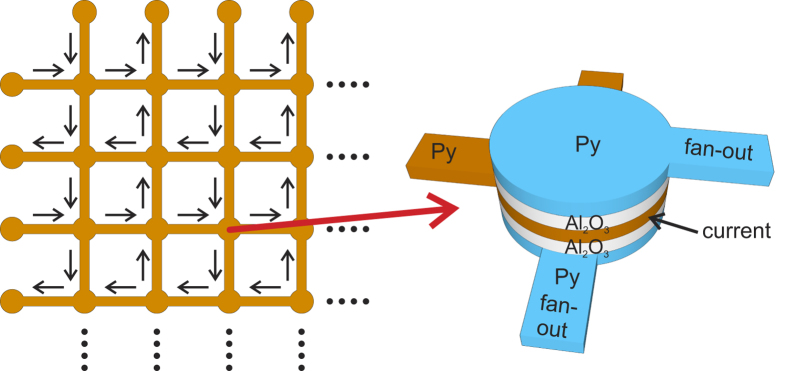
Memory and logic network based on the 3-terminal logic gate. The two-dimensional network array consists of nodes and channels. Each node serves as both a memory unit and a logic gate, i.e., the logic operation occurs at the node. The topological computation resulting between two neighbouring memory bits is directly written into the target node. Each node is a ‘two-in, two-out’ structure as illustrated on the right. The ‘two-in’ terminals constitute the logic gate, which stores the computed binary information. The storage layer is sandwiched between the two symmetrical, aperture-based fan-out structures described in Fig. 5. The arrows denote the directions of the data flow within the network.

Moreover, the gate terminal (see Fig. 6a) can also be extended such that the result of the logic operation stored in the gate *G* is fed to successive elements, utilising the same working principle. As shown in Fig. 7, for each gate terminal, two symmetric fan-out structures can be attached to the top and bottom of the gate terminal. This forms a ‘two-in, two-out’ node, which can be expanded two-dimensionally, forming a network array. The architecture is shown in Fig. 7. Each node serves as a nonvolatile memory bit, encoded by the topology. The information at each node can be ‘copied’ and ‘pasted’ to any of other nodes within the network by using the fan-out mechanism. They follow the data flow indicated by the arrows in the figure. Therefore, direct information transfer among all storage nodes is realised. More importantly, logic computations between the neighbouring ‘two-in’ memory bits can be directly performed and then stored. As the network allows for free data flow, in principle all memory bits can communicate with one another in the network. This not only unifies memory and logic elements, but also allows for highly efficient computing.

### Summary

To summarize, the direct spin communication and computation scheme has significant advantages over traditional, electrically-operated logic gates in which the stored information has to be read out electrically and transferred to a specific logic gate to implement operations. Further, in traditional devices, the outputs of the logic operations have to be transferred back to the magnetic memory via a writing procedure. This is both time- and energy-consuming. The self-computing device, on the other hand, allows stored information to directly travel and communicate, with results of such computations being stored in the output bit – all within a non-volatile framework.

## Methods

### Micromagnetic simulation

Micromagnetic simulations were carried out using a commercial finite-difference Landau-Lifshitz-Gilbert (LLG) equation simulator^34^. The simulation object is defined on a two-dimensional grid with a cell size of 2 × 2 nm^2^. The thickness is kept at 2 nm. Permalloy with a saturation magnetisation of 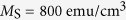, exchange stiffness 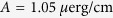, and uniaxial anisotropy constant 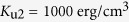 was assumed. The gyromagnetic ratio *γ*_0_ was chosen to be 17.6 MHz, while the damping constant *α* was 0.02 to increase the dynamical effects during the computation. Minor influences on the general properties of the VA dynamics were found when *α* was varied significantly, while the effect on the travelling distance was more pronounced. For the simulations, a VA pair is created as the initial domain pattern using Eq. (9). In principle, it can also be generated using the aperture described in Fig. 5.

## Additional Information

**How to cite this article**: Zhang, S. *et al.* Topological computation based on direct magnetic logic communication. *Sci. Rep.* **5**, 15773; doi: 10.1038/srep15773 (2015).

## Supplementary Material

Supplementary Information
