# Supplementary material for: Topological computation based on direct magnetic logic communication
Source: Sci Rep. 2015 Oct 28;5:15773. doi: 10.1038/srep15773 (PMC4623769; doi:10.1038/srep15773)
Supplement: Supplementary Information [file srep15773-s1.pdf]

Supporting Online Material for

Topological computation based on direct magnetic logic communication

Shilei Zhang,[1] Alexander A. Baker,[1]

Stavros Komineas,[2] and Thorsten Hesjedal[1*]

[1]Department of Physics, Clarendon Laboratory, University of Oxford, Oxford, OX1 3PU, UK
[2]Department of Mathematics and Applied Mathematics, University of Crete,
71409 Heraklion, Crete, Greece

Here, we present a short summary of logic gates, starting with the basic logic gates. Next, a short summary of the material implication gate is given, which is naturally implemented with the topological computing scheme introduced in this work .

1. Logic gates

Figure 6 in the main text summarizes the various logic operations possible using topological objects. By joining two 2-terminal fan-out structures in a common terminal gate, different logic gates can be realized, depending on the geometrical dimensions of the two basic structural elements. The key idea is to make use of the fact that, when a vortex-antivortex (VA) pair is sent traveling down such an element, the VA pair either completes the journey, or decays in to a single vortex. As a result of the arrival time-dependent interaction of the vortex patterns at the gate, the behavior of an XOR or NIMP gate is obtained, which will be explained in more detail below. The 3-terminal structure, shown in Fig. 6d in the main text, functions as a controlled IMP gate where the control bit determines whether the gate functions as an IMP, or a NOT-IMP (= NIMP).

In general, for performing all possible logic operations, also known as achieving functional completeness, a set of logic gates is needed, each of which implement a Boolean logic function. A short summary of logic gates and computation can be found, e.g., in Ref. [S1].

In recent years, with the development of memristor-based logic [S2], another logic concept was brought back into the center of attention, the 'material implication', or IMP gate, which was first introduced in Whitehead and Russell's book *Principia Mathematica* in 1910 [S3]. The IMP operation, in conjunction with the FALSE operation, also forms a computationally complete logic basis [S4], as will be discussed in more detail below.

1.1. Basic logic gates

A computationally complete logic basis is needed to obtain all possible truth tables. The truth tables showing all functional values of the basic logic gates on each of their inputs *A* and *B*, is shown below in Table S1. A TRUE input is represented by a logic '1' and a FALSE by a logic '0'. The basic logic gates comprise AND, OR, NOT, the NOT-AND (= NAND) and NOT-OR (= NOR). In case of the OR gate, the ambiguity to treat the case of two TRUE inputs is resolved by defining the normal OR gate as one where at least one of the inputs has to be TRUE for the result to be true, whereas the exclusive OR, or XOR's output is only then TRUE when only one input is TRUE. The XNOR is consequently the NOT-XOR.

Inputs		Output						
A	***B***	**AND**	**OR**	**NOT**	**NAND**	**NOR**	**XOR**	**XNOR**
0	0	0	0	1	1	1	0	1
0	1	0	1	1	1	0	1	0
1	0	0	1	0	1	0	1	0
1	1	1	1	0	0	0	0	1

Table S1: Basic logic operations on the inputs A and B. Note that the NOT operation is only on a single input, A.

A computationally complete logic basis is given by the set comprising the AND and NOT gates. For obvious reasons, working with the smallest possible complete set has advantages for devices. A complete set is already realized by the NAND or NOR gates alone, making them the preferred choice for logic devices. However, this choice of fundamental gates used in digital electronics is not unique. An example of an alternative approach is briefly summarized in the next Section.

1.2. Material implication gate

The material implication, or IMP gate, was introduced by Whitehead and Russell in their seminal book *Principia Mathematica*, first published in 1910 [S3]. The operation *A* IMP *B* means *A* implies *B* and the corresponding truth table is shown in Table S2. The IMP gate, in conjunction with a FALSE gate, is once again computationally complete [S5]. These logic concepts attracted a lot of attention as they are naturally realized in devices comprising of memristive elements. The interested reader is referred to the work of Borghetti *et al.* [S4], and references therein, for a detailed discussion of the topic. As demonstrated in the main text, the topological computing scheme introduced by us is also inherently showing the same logic functionality of the material implication, making it a universal logic platform for device applications.

Inputs		Output						
A	*B*	*A* IMP *B*	*B* IMP *A*	*A* NIMP *B*	*B* NIMP *A*	TRUE<br><br>*A* IMP *A*	*A* NOR *B*<br><br>((*A* IMP *0*)<br><br>IMP *B*) IMP *0*	*A* NAND *B*<br><br>*A* IMP (*B* IMP *0*)
0	0	1	1	0	0	1	1	1
0	1	1	0	0	1	1	0	1
1	0	0	1	1	0	1	0	1
1	1	1	1	0	0	1	0	0

Table S2: Truth table of the computationally universal IMP gate, in all combinations of *A* IMP *B*, *B* IMP *A*, and the NOT-IMP (= NIMP) gates, and selected examples of equivalent operations of basic logic gates represented by IMP gates. A complete list can be found in the Supplementary Material to Ref. [S4].

References and Notes

S1. Horowitz, P. & Hill, W. The art of electronics. Cambridge University Press (2015).

S2. Strukov, D. B., Snider, G. S., Stewart, D. R. & Williams, R. S. The missing memristor found, *Nature* **453**, 8083 (2008).

S3. Whitehead, A. N. & Russell, B. Principia Mathematica to ∗56. Cambridge Mathematical Library (1997).

S4. Borghetti, J., Snider, G. S., Kuekes, P. J., Yang, J. J., Stewart, D. R. & Williams, R. S. ‘Memristive’ switches enable ‘stateful’ logic operations via material implication, *Nature* **464**, 873-876 (2010).

S5. Lehtonen, E. & Laiho, M. Stateful implication logic with memristors, in 2009 IEEE/ACM International Symposium on Nanoscale Architectures, pp. 33-36.
